# Attenuating Sulfidogenesis in a Soured Continuous Flow Column System With Perchlorate Treatment

**DOI:** 10.3389/fmicb.2018.01575

**Published:** 2018-07-26

**Authors:** Anna L. Engelbrektson, Yiwei Cheng, Christopher G. Hubbard, Yong T. Jin, Bhavna Arora, Lauren M. Tom, Ping Hu, Anna-Lena Grauel, Mark E. Conrad, Gary L. Andersen, Jonathan B. Ajo-Franklin, John D. Coates

**Affiliations:** ^1^Energy Biosciences Institute, University of California, Berkeley, Berkeley, CA, United States; ^2^Department of Plant and Microbial Biology, University of California, Berkeley, Berkeley, CA, United States; ^3^Lawrence Berkeley National Laboratory, Earth Sciences Division, Berkeley, CA, United States

**Keywords:** perchlorate reduction, petroleum microbiology, souring, sulfate reduction, sulfur

## Abstract

Hydrogen sulfide production by sulfate reducing bacteria (SRB) is the primary cause of oil reservoir souring. Amending environments with chlorate or perchlorate [collectively denoted (per)chlorate] represents an emerging technology to prevent the onset of souring. Recent studies with perchlorate reducing bacteria (PRB) monocultures demonstrated that they have the innate capability to enzymatically oxidize sulfide, thus PRB may offer an effective means of reversing souring. (Per)chlorate may be effective by (i) direct toxicity to SRB; (ii) competitive exclusion of SRB by PRB; or (iii) reversal of souring through re-oxidation of sulfide by PRB. To determine if (per)chlorate could sweeten a soured column system and assign a quantitative value to each of the mechanisms we treated columns flooded with San Francisco bay water with temporally decreasing amounts (50, 25, and 12.5 mM) of (per)chlorate. Geochemistry and the microbial community structure were monitored and a reactive transport model was developed, Results were compared to columns treated with nitrate or untreated. Souring was reversed by all treatments at 50 mM but nitrate-treated columns began to re-sour when treatment concentrations decreased (25 mM). Re-souring was only observed in (per)chlorate-treated columns when concentrations were decreased to 12.5 mM and the extent of re-souring was less than the control columns. Microbial community analyses indicated treatment-specific community shifts. Nitrate treatment resulted in a distinct community enriched in genera known to perform sulfur cycling metabolisms and genera capable of nitrate reduction. (Per)chlorate treatment enriched for (per)chlorate reducing bacteria. (Per)chlorate treatments only enriched for sulfate reducing organisms when treatment levels were decreased. A reactive transport model of perchlorate treatment was developed and a baseline case simulation demonstrated that the model provided a good fit to the effluent geochemical data. Subsequent simulations teased out the relative role that each of the three perchlorate inhibition mechanisms played during different phases of the experiment. These results indicate that perchlorate addition is an effective strategy for both souring prevention and souring reversal. It provides insight into which organisms are involved, and illuminates the interactive effects of the inhibition mechanisms, further highlighting the versatility of perchlorate as a sweetening agent.

## Introduction

The interplay between anaerobic hydrocarbon degradation by sulfate reducing bacteria (SRB) and the accompanying hydrogen sulfide (H_2_S) production in oil rich environments is critical to industrial sustainability and facility management. *In-situ* biogenic H_2_S production in oil and gas reservoirs (known as souring) represents a significant threat to both the operator personnel and facilities integrity. H_2_S is toxic, explosive, and highly corrosive. Through occupational exposure, it is the primary cause of industrial gas inhalation deaths in the United States and is the underlying cause of corrosion resulting in infrastructure and pipeline failure with associated annual costs in excess of $90B annually^1^.

For nearly a century reservoir souring has been recognized as a substantial problem for the oil industry (Bastin et al., 1926). Water flooding as a secondary method of recovery is the biggest contributing factor to reservoir souring. This process involves the continuous injection of large volumes of water to displace the oil from the rock matrices. In offshore facilities seawater is used, which contains approximately 28 mM sulfate and an active indigenous microbial community with substantial SRB populations (Youssef et al., 2009). Cooling in the near well injection zone as a result of cold (∼4°C) seawater injection creates an active mixing zone and a thermal gradient that is conducive to microbial activity, even in reservoirs that are normally too hot for SRB survival. The presence of labile volatile fatty acids, often naturally present in the formation water, in addition to labile crude oil components vulnerable to SRB catabolism, stimulate the activity of these organisms with resultant rampant sulfide production (Gieg et al., 2011).

Nitrate addition alone or combined with biocides has historically been the primary strategy to inhibit souring (Myhr et al., 2002; Voordouw et al., 2009; Gieg et al., 2011). Nitrate reduction is thermodynamically favorable over sulfate reduction and nitrate reducing microorganisms biologically remove sulfide by re-oxidation to sulfate or elemental sulfur (Greene et al., 2003; Gieg et al., 2011). Recently, a significant body of evidence has grown to suggest that nitrite production from incomplete nitrate reduction serves as a direct inhibitor of SRB and is the primary mechanism by which nitrate addition controls souring (Greene et al., 2003; Hubert and Voordouw, 2007). However, nitrate treatment can also cause corrosion and is not universally successful or predictable as a remediation strategy for souring (Hubert et al., 2005; Engelbrektson et al., 2014).

Recent research indicates that perchlorate shows promise as a novel alternative inhibitor of sulfate reduction (Engelbrektson et al., 2014; Gregoire et al., 2014; Carlson et al., 2015; Mehta-Kolte et al., 2017). Postgate (1952) first suggested that (per)chlorate could inhibit the model SRB *Desulfovibrio*. However, the broad-spectrum effectiveness and underlying mechanism of (per)chlorate inhibition was not investigated in detail until recently (Carlson et al., 2015). Previous work demonstrated perchlorate to be an effective inhibitor of the onset of souring in a advective packed column system and concluded that perchlorate has greater potency than nitrate at equal concentrations and stimulated different, potentially more favorable, sulfur cycling (Engelbrektson et al., 2014; Gregoire et al., 2014; Mehta-Kolte et al., 2017). Modeling of the column experiment in the Engelbrektson et al. (2014) study suggested that for perchlorate treatment the most important effector was biocompetitive exclusion by perchlorate reducing bacteria (PRB) activity, while for the nitrate treatment the primary effector was nitrite produced from incomplete nitrate reduction (Cheng et al., 2016). Carlson et al. (2015) found that (per)chlorate and nitrate are both specific inhibitors of sulfate reduction, thus inhibiting growth and sulfide production in both enrichment and pure cultures of SRB, and that nitrate and (per)chlorate have a common mode of action as competitive inhibitors of the prerequisite ATP-sulfurylase enzyme, with (per)chlorate being more effective at lower concentrations. However, at field application rates and resultant concentrations, this aspect of inhibition is unlikely to be of significance for either compound.

Although (per)chlorate has shown effectiveness at inhibiting the onset of souring it has not yet been clearly demonstrated that it can also be effective as a sweetening (souring reversal) agent in a soured system. However, two recent studies demonstrated that all perchlorate reducing organisms have the ability to innately oxidize hydrogen sulfide to elemental sulfur (Gregoire et al., 2014; Mehta-Kolte et al., 2017), suggesting that (per)chlorate shows promise as a sweetening agent because stimulating PRB activity should both inhibit SRB and stimulate the conversion of already present H_2_S to elemental sulfur. Here, we investigate the use of perchlorate to sweeten a soured column system and test the concentration of perchlorate needed to keep a sweetened system from re-souring by stepping down the inhibitor concentration until re-souring is seen. We also investigate the specific mechanisms underlying the effectiveness of perchlorate treatment at each treatment concentration through the development and testing of a reactive transport model.

## Materials and Methods

### Column Setup

Twelve 1 L advective up-flow columns were packed with a pre-soured mixture of San Francisco bay water, San Francisco bay sediment, yeast extract, and sand (Supplementary Figure S1). All columns were initially flooded at 0.17 mL/min (estimated 50 h hydraulic retention time) with autoclaved San Francisco Bay water containing 2 g/L yeast extract, to serve as an electron donor, and allowed to stabilize for 21 days. Once stable sulfide production was observed in all columns inhibitor treatment (sodium nitrate, sodium chlorate, or sodium perchlorate) was initiated (day 0) in triplicate. One triplicate column set was left untreated. Treatment phases and lengths are summarized in Supplementary Table S1. Initially, referred to as phase 1 throughout, treatment was applied at 51.4 ± 3.79 mM (mean ± 1σ, *n* = 3, average of all treatments). At day 102, this treatment was reduced to 23.48 ± 3.09 mM (phase 2) and at day 221 it was further reduced to 12.16 ± 1.48 mM (phase 3). During phase 1 of treatment there was a 14-day shut in period where no flow was applied to the columns.

### Geochemical Measurements

Geochemistry samples were collected by pulling liquid from the effluent port of the column with a syringe and filtering through a 0.2 μm nylon syringe filter. Sulfide concentrations were measured using a modified Cline assay (Cline, 1969; Engelbrektson et al., 2014). Briefly, samples were processed immediately and diluted with anaerobic deionized water to bring them into a measurable range, mixed with assay reagents and read at 660 nm on a Varian Cary 50 Bio spectrophotometer equipped with a Cary 50 MPR microplate reader. Following this, sulfide was removed from the remaining sample by adding FeCl_3_ to bind with the sulfide and NaOH to precipitate out the iron sulfide complexes. Samples were then centrifuged and re-filtered before storage at 4°C. Sulfate, chlorate, and perchlorate concentrations were measured using ion chromatography on an Dionex ICS-1500 equipped with a Thermo Scientific Dionex IonPac AS25 Hydroxide-Selective Anion-Exchange Column using a 35 mM sodium hydroxide flow rate of 1 mL/min. Nitrate was measured using a Dionex ICS-2100 equipped with Thermo Scientific Dionex IonPac AS16 Hydroxide-Selective Anion-Exchange Column with a 25–65 mM potassium hydroxide gradient flow at a rate of 1 mL/min.

### Isotopic Analyses

Isotope analysis of dissolved sulfide (δ^34^S) and dissolved sulfate (δ^34^S and δ^18^O) were performed on 20–30 mL samples collected from the effluent port at various points throughout the study. Influent samples were also collected for isotope analysis throughout the study (δ^34^S and δ^18^O of sulfate, δ^18^O of water). Dissolved sulfide was precipitated out by adding excess zinc acetate to a 0.2 micron nylon filtered sample. The precipitate was then washed with 3% ammonium hydroxide and deionized water before drying at 50°C. The supernatant was re-filtered before precipitating sulfate by first acidifying with hydrochloric acid (to prevent carbonate precipitation) and then adding excess barium chloride. The resulting barium sulfate precipitate was washed with deionized water before drying at 105^°^C. Sulfur and sulfate-oxygen isotope ratios were measured using GV Isoprime isotope ratio mass spectrometer along with the Eurovector Elemental Analyser (EuroEA3028-HT) in the Laboratory for Environmental and Sedimentary Isotope Geochemistry (LESIG) at Department of Earth and Planetary Science, University of California at Berkeley. Isotope ratios are reported in standard delta notation relative to international standards, e.g., δ^34^S = (R_sample_/R_std_ - 1) × 1000, where R = ^34^S/^32^S, and the value is reported in per mil units (‰) relative to the Canyon Diablo Troilite standard (R_std_ = 0.0441216). Typical instrument reproducibility (1σ) as assessed on reference materials is ±0.2‰ for δ^34^S, ±0.3‰ for δ^18^O-sulfate, and ±0.03‰ for δ^18^O-water.

### Microbial Community

To follow changes in community structure throughout the experiment, sediment samples were collected from the column near the outlet port (Pre-treatment – day 0, phase 1 – days 10 and 59, phase 2 – days 130 and 178, and phase 3 – day 263), frozen immediately on dry ice, and stored at -80°C. For RNA isolation the sample tubes were warmed by hand until loose enough to shake into a sterile whirl-pack bag (Nasco, Fort Atkinson, WI, United States). The bags were cooled on dry ice, wrapped in a paper towel, and crushed with a hammer. 0.5 g of sample were weighed into 2 mL Lysing Matrix E tubes (MP Biomedicals, Solon, OH, United States) and extracted as previously described (DeAngelis et al., 2010) using a modified CTAB extraction buffer consisting of equal volumes of 0.5 M phosphate buffer (pH 8) in 1M NaCl and 10% hexadecyltrimethylammonium bromide (CTAB) in 1 M NaCl. Briefly, tubes containing 0.5 g of sample, 0.5 mL of modified CTAB extraction buffer, 50 uL of 0.1 M ammonium aluminum sulfate and 0.5 mL of phenol: chloroform: isoamyl alcohol (25:24:1) were bead-beat at 5.5 m/s for 45 s in a FastPrep instrument (MP Biomedicals, Solon, OH, United States). Following bead-beating, tubes were centrifuged at 16,000 × *g* for 5 min at 4°C. The supernatant was transferred to a new tube containing an equal volume of chloroform: isoamyl alcohol (24:1), vortexed, and centrifuged again. The supernatant was transferred into a new tube containing 1 mL of Peg 6000 solution and 1 uL of linear acrylamide and incubated at room temperature for 2 h. Each sample was extracted a second time by adding 0.5 mL of modified CTAB extraction buffer to the original Lysing Matrix E tubes and repeating the steps from bead-beating onwards. The first and second extractions were centrifuged at 16,000 × *g* for 10 min at 4°C. The pellets (two per sample) were washed with 0.5 mL of cold 70% ethanol, dried, and combined in 50 uL of RNase-Free water. Purification was carried out using the AllPrep DNA/RNA Mini Kit (Qiagen, Valencia, CA, United States) with on-column DNAse digestion using the RNase-Free DNase Set (Qiagen, Valencia, CA, United States) according to manufacturer’s instructions. RNA was eluted in 30 uL RNase-Free water. Concentrations were assessed by Qubit fluorimeter (Invitrogen, Carlsbad, CA, United States).

All solutions and plastics were either certified RNase free or treated with 0.1% diethyl pyrocarbonate. Each set of extractions included a DNA and RNA extraction blank (EB). All DNA EBs were amplified using 16S bacterial primers 27F and 1492R (Hazen et al., 2010). A single 25 uL PCR reaction was carried out as previously described (Hazen et al., 2010) with the following modifications: 200 nM of each primer, 5 uL of each DNA EB as template, and 50°C annealing temperature. 5 uL of PCR product was loaded onto a 2% agarose gel to check for contamination. All RNA extracts (including RNA EBs) were also amplified using the same 16S primers to check for DNA contamination. PCR was carried out as above using 1 uL as template. 10 uL of PCR product was loaded onto a 2% agarose gel to check for DNA contamination. Samples with DNA contamination were re-digested with DNase and re-checked via PCR until they were confirmed DNA free. All DNA and RNA EBs were confirmed to be clean before proceeding with downstream processing.

Fifty nanograms or 10 uL of RNA was converted to single-stranded cDNA using SuperScript II reverse transcriptase (Invitrogen, Carlsbad, CA, United States) and a custom primer mix targeting the 16S rRNA gene, made from equal volumes of 9uM 27F, 1492R (Hazen et al., 2010), and rD1 (Weisburg et al., 1991). Freshly synthesized cDNA was used as template for PCR.

The 16S rRNA gene was amplified using universal bacterial primers 27F and 1492R (Hazen et al., 2010). 2 uL of freshly synthesized cDNA was used as template for PCR. Each PCR reaction contained 1× Ex Taq buffer (Takara Bio Inc., Japan), 0.025 U/uL Ex Taq polymerase, 0.8 mM dNTP mixture, 1.0 ug/uL BSA, 200 nM of each primer, and either DNA or freshly synthesized cDNA. Samples were amplified in four replicate 25 uL reactions spanning a range of annealing temperatures. PCR conditions were 95°C (3 min), followed by either 25 cycles (DNA) or 30 cycles (cDNA) of 95°C (30 s), 50–56°C (30 s), 72°C (2 min), followed by a final extension 72°C (10 min). PCR product from each 4-temperature gradient was pooled and 10 uL (cDNA) and 3 M NaAc was added to adjust the pH. Samples were purified with the MinElute PCR Purification Kit (Qiagen, Valencia, CA, United States) and eluted in 25 uL of elution buffer. 1 uL of purified PCR product was quantified on a 2% agarose gel using Low-Range Quantitative DNA Ladder (Invitrogen, Carlsbad, CA, Unites States).

For cDNA samples, 300 ng or 22 uL (if < 300 ng) of bacterial PCR product was hybridized to each array following previously described procedures (Hazen et al., 2010). Briefly, bacterial PCR product and a custom spike mix containing amplicons of known concentration were combined, fragmented to 50–200 bp using DNAse I (Invitrogen, Carlsbad, CA, United States), biotin labeled, and hybridized overnight at 48°C and 60 rpm. The arrays were washed, stained, and scanned as previously described. Data from the resulting CEL files were processed through PhyCA using the same Bacterial Stage1 and Stage2 cutoffs as previously described (Piceno et al., 2014). The intensity data were rank-normalized using a custom R script where intensity values were ranked (ordered from lowest to highest intensity and assigned a corresponding number). Data are included as Supplementary Table S2.

Statistical analyses on the OTU data were performed using Primer 7 (Clarke and Gorley, 2015). All data were standardized, square root transformed, and a Bray Curtis similarity matrix was created. NMDS plots were then created using this similarity matrix (Clarke, 1993). Similarity clustering on the plots (circles) were created using hierarchical clustering using group average to form a dendogram with a SIMPROF test for significant clusters (Clarke et al., 2008). All clusters circled on the NMDS plots were significant by SIMPROF. Means NMDS plots were created by averaging the replicate samples and creating a Bray Curtis similarity matrix from the averaged values. Trajectories were plotted on these plots using the trajectory tool in Primer 7. Similarity percentage (SIMPER) was used to determine the OTUs contributing to the top 10% of the differences between various groupings. The average abundance in the SIMPER output for each OTU was subtracted from the comparison group’s value and positive values (indicating enrichment in that condition) were separated from the negative values (indicating inhibition in that condition). These values were then summed by family or phylum (class for Proteobacteria) and used to create enrichment and inhibition graphs in Excel.

### Reactive Transport Modeling

CrunchTope (Steefel and Maher, 2009; Druhan et al., 2012, 2013, 2014), a reactive transport code, was used to develop a mechanistic model of the coupled biochemical and flow processes that occurred in the perchlorate-treated columns. A general reactive transport equation for chemical species, *i* (Steefel et al., 2014):

(1) $$\frac{\partial (\varphi S_{L}C_{i})}{\partial t}=\nabla \cdot \left(\varphi S_{L}D_{i}\nabla C_{i}\right)-\nabla \cdot (qC_{i})-\sum_{j=1}^{Nj}v_{ij}R_{j}-\sum_{g=1}^{Ng}v_{ig}R_{g}-\sum_{m=1}^{Nm}v_{im}R_{m}$$

where, the term on the left-hand side is the accumulation term and the terms on the right-hand side are diffusion, advection, and reaction terms (*R_j_*: aqueous phase reactions, *R_g_*: gas reactions, *R_m_*: mineral reactions), respectively. *ϕ* is porosity, *S_L_* is liquid saturation, *C_i_* is concentration (mole per kilogram_water_), *D* is the diffusion coefficient (square meter per second), and *q* is the Darcy flux (meter per second).

In CrunchTope, dynamics relating bacterial growth and energetics follow the conceptual framework as described in McCarty and Rittmann (2001). In the environment, bacteria (B) mediate the reaction between an electron donor and an electron acceptor (i.e., sulfate and perchlorate in this study) to derive energy for growth and maintenance. Rates of microbially mediated reactions are described as follows:

(2) $$r=\mu [\text{B}]K_{T}r=\mu [\text{B}]K_{T}$$

where *r* (mole per kilogram_water_ per day) is rate of the reaction as mediated by B (represented as C_5_H_7_O_2_N), μ (mole per mole_C5H7O2N_ per day) is the maximum-specific utilization rate. Kinetic constraints on the reaction rate by electron acceptors/donors and inhibitors are mathematically represented as:

(3) $$K_{T}=\frac{\left[\text{eDonor}\right]}{[\text{eDonor}]+K_{\text{eDonor}}\;}\frac{\left[\text{eAcceptor}\right]}{[\text{eAcceptor}]+K_{\text{eAcceptor}}}\frac{K_{\text{Inhibitor}}}{[\text{Inhibitor}]+K_{\text{Inhibitor}}}$$

*K*_eDonor/eAcceptor_ (mole per kilogram_water_) is the half saturation (affinity constant) of the electron donor/acceptor, while *K*_inhibitor_ (mole per kilogram_water_) is the inhibition constant.

### Model Setup and Simulations

The one-dimensional reactive transport simulations were conducted over a simulation domain of 100 grids (resolution = 0.00254 m). Porosity was set at 0.32. A constant flow velocity of 0.05695 m day^-1^ was prescribed. Initial and influent concentrations of chemical species can be found in Supplementary Table S3. The three known microbiological mechanisms by which perchlorate inhibits sulfate reduction are: (1) indirect inhibition of the SRB through competition with heterotrophic PRB (hPRB) for electron donors, (2) direct inhibition of SRB activities by perchlorate (Postgate, 1952; Baeuerle and Huttner, 1986), and (3) perchlorate reduction linked to sulfide oxidation (PRSO) of the PRB (Engelbrektson et al., 2014; Gregoire et al., 2014; Mehta-Kolte et al., 2017). Mehta-Kolte et al. (2017) demonstrated that PRB mediate both heterotrophic perchlorate reduction (HPR) and PRSO (to elemental sulfur, **Table 1**). The PRSO pathway is preferred, however, no growth is observed. PRB grow during HPR, a metabolism that is only sustainable in the absence of sulfide. In the model, the PRB population mediates both pathways, with a sulfide inhibition constant applied to HPR (**Table 2**). Yeast extract (2 g/L) is used as a multivariate non-selective electron donor in the experiment. In an anaerobic bottle experiment with San Francisco Bay water, sediment and yeast extract, 1 g/L yeast extract reduced 18.2 mM sulfate, equivalent to ∼20 mM of acetate according to reaction stoichiometry (**Table 1**). Therefore in our simulation, 2 g/L of yeast extract is represented by 40 mM acetate.

**Table 1:** Microbial and iron-sulfide reactions modeled.

Microbial Reactions	
**1.**	**Sulfate reduction** (SO_4_^2-^- > H_2_S_(aq)_) (fs = 0.08, fe = 0.92)
	0.115SO_4_^2-^ + 0.125DOC + 0.004NH_3_ + 0.23H_2_O + 0.01H^+^ 0.004C_5_H_7_O_2_N_SRB_ + 0.23HCO_3_^-^ + 0.115HS^-^

**2.**	**Heterotrophic perchlorate reduction** (ClO_4_^2-^ - > Cl^-^) (fs = 0.45, fe = 0.55)
	0.05625ClO_4_^2-^ + 0.125 DOC + 0.0275NH_4_^+^ + 0.0525H_2_O 0.0275C_5_H_7_O_2_N_PRB_ + 0.2475HCO_3_^-^ + 0.05625Cl^-^ + 0.2475H^+^

**3.**	**Perchlorate reduction sulfide oxidation** (HS^-^ - > S_(aq)_) (fs = 0.0, fe = 1.0)
	0.125ClO_4_^-^ + 0.5H^+^ + 0.5H^32^S^-^ 0.125Cl^-^ + 0.5H_2_O + 0.5S_(aq)_

**4.**	**Iron-sulfide reactions**
	**a.** Fe^2+^ + H_2_S_(aq)_ ↔ FeS_(am)_ + H^+^
	**b.** Fe(OH)_3(s)_ + 0.5H_2_S_(aq)_ + 2.5H^+^ ↔ Fe^2+^ + 0.5S_(s)_ + H_2_O + 2.0OH^-^

*Values of *fs* and *fe* are determined by the free energy values of electron donors and acceptors involved in the microbial reaction (McCarty and Rittmann, 2001). *fs* and *fe* relate electron donor, electron acceptor and cell synthesis half equations following: *R* = *fe*⋅*R_*a*_* + *fs*⋅*R_*c*_* - *R_*d*_*. Where *R* is the resulting equation, *R_*a*_* is the electron acceptor half equation, *R_*d*_* is the electron donor half equation, and *R_*c*_* is the cell synthesis half equation.*

**Table 2:** Kinetic parameters of reactions in **Table 1**.

Microbial reactions^#^	*μ* [mol (mol cell)^-1^ day^-1^]	*K*_acceptor_ (mol kgw^-1^)	*K*_donor_ (mol kgw^-1^)
1	194^(a)^	1.0 × 10^-3(a)^	1.0 × 10^-3(a)^
2	172^(a)^	0.5 × 10^-3^	0.5 × 10^-3^
3	176^(a)^	1.0 ×10^-2^	1.0 × 10^-2^

**Mineral dissolution and precipitation**	**log*k* (mol/m^2^/s)**	**log*K*_eq_**	

4a	-7.0^(b)^	3.5^(b)^	
4b	-10.0	-19.6^(b)^	

**Microbial reactions**	**Inhibitor**	***K*_inhib_ (mol kgw^-1^)**	

1	Perchlorate	2.0 × 10^-3(c)^	
2	Sulfide	2.6 × 10^-3(d)^	

*^#^Rxn number from **Table 1**. (a) Values from Cheng et al. (2016). (b) Values from Li et al. (2009), Druhan et al. (2012, 2014), and Cheng et al. (2016). (c) Range: 2.0 × 10^-*3*^ – 30.0 × 10^-*3*^ M based on Carlson et al. (2015). (d) Value from Gregoire et al. (2014) and Mehta-Kolte et al. (2017).*

Models are effective tools in elucidating the importance of different microbially mediated pathways under varying geochemical conditions (e.g., sulfide concentrations). For perchlorate treatment, the following scenarios were explored to tease out the effect of different mechanisms to sulfide production:

Case 1: (*base case simulation*): This implements all mechanisms by which perchlorate inhibits sulfide production.
Case 2: Base case simulation, except that direct inhibitory effect of perchlorate on sulfate reduction was not considered. The inhibition term in Eq. 1 was removed and the rest of the parameters were same as in the base case.
Case 3: Base case simulation, except that HPR was not considered. Reactions 2 from **Table 1** were removed.
Case 4: Base case simulation, except that perchlorate-dependent sulfide oxidation was removed. Reactions 3 from **Table 1** were removed.

Values of kinetic parameters followed previously published values. For example, log*K_eq_* for the iron-sulfide reactions (as defined in **Table 1**) are 3.5 and -19.6, respectively, following those from Li et al. (2009) and Druhan et al. (2014). In the case that a range of published values exists for a particular model parameter, the model parameter was fine tuned (within the published range) such that the simulation (i.e., base case) matched the observed data. For example, the published values for the inhibition constant for the inhibition of sulfate reduction by perchlorate is 2.0 × 10^-3^ – 30.0 × 10^-3^ M (Carlson et al., 2015). Through calibration, a value of 2.0 × 10^-3^ was applied to the model. Parameter values for microbial reactions can be found in **Tables 1, 2**.

## Results

### Geochemical Results

**FIGURE 1:**
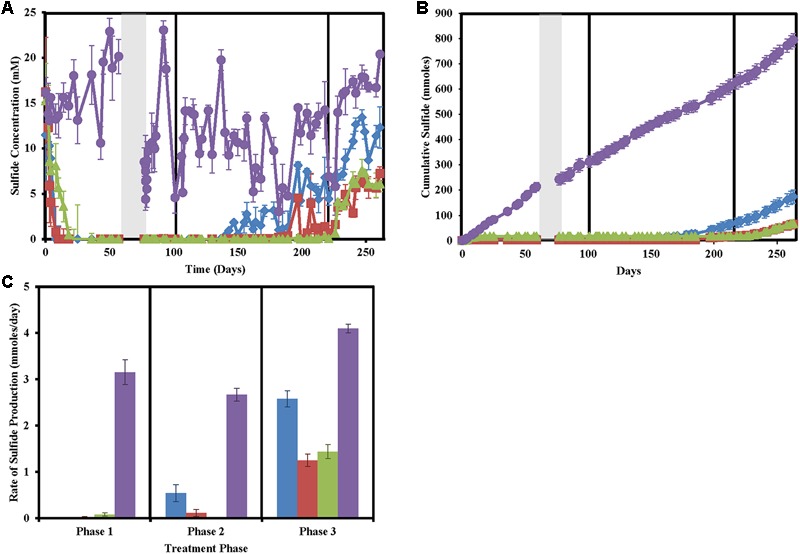
Sulfide production in the column sets over time for nitrate-treated columns (blue diamonds/bars), chlorate (red squares/bars), perchlorate (green triangles/bars), no treatment (purple circles/bars), and influent (black stars). Phases of treatment are represented by boxes with the first being 50 mM, followed by 25 mM, and 12 mM. The shut in period is represented by a gray bar. All points are averages of triplicate columns with error bars representing SD of the triplicate samples. **(A)** Effluent sulfide concentrations over time. **(B)** Cumulative sulfide measured in the effluent over time. **(C)** Rates of sulfide production during each treatment phase.

After a suitable equilibrium period prior to any treatment, column effluent sulfide concentrations averaged at 14.08 ± 2.76 mM (**Figure 1**). In phase 1 of treatment, both nitrate and chlorate-treated columns achieved full sweetening (no further sulfide production) by day 10. Perchlorate-treated columns achieved the same result by day 18 (**Figures 1A,B**). After the 2-week shut in period, during phase 1, sulfide levels in all untreated columns quickly rebounded while sulfide concentrations in the effluent of the treated columns remained below detection. In phase 2 of the column operation, when the concentration of the (per)chlorate and nitrate was reduced by approximately 50% to 23.48 ± 3.09 mM, both the nitrate and chlorate columns began producing sulfide (**Figures 1A,B**). Nitrate-treated columns re-soured by day 140 (38 days after the treatment concentration was reduced) and souring temporally increased for the remainder of the column operation. In contrast, sulfide production in the chlorate-treated columns was intermittent from day 189 (87 days after treatment concentration was reduced), 49 days after steady re-souring onset occurred in the nitrate-treated columns (**Figures 1A,B**). Juxtaposed to both the nitrate- and chlorate-treated columns, perchlorate-treated columns did not re-sour in this treatment phase, and no measurable sulfide was detected in the effluent. In phase 3, immediately after the treatment concentration was reduced by a further 50% to 12.16 ± 1.48 mM, steady re-souring was observed in both the perchlorate- and chlorate-treated columns. Cumulative sulfide production data throughout the operation of the columns (**Figure 1B**) clearly highlights the differences in the souring treatment effectiveness. Furthermore, calculation of the sulfide production rate in phase 3 of the treatment regime indicates that while the nitrate-treated columns had a lower rate of souring (2.58 ± 0.17 mmoles/day) than the control columns (4.10 ± 0.09 mmoles/day), it was still significantly higher than either the perchlorate (1.44 ± 0.15 mmoles/day) or chlorate-treated columns (1.25 ± 0.13 mmoles/day; ANOVA: *P* < 0.0001, *F* = 194.3; Tukey multiple comparisons test: only non-significant comparison is perchlorate versus chlorate treatments, **Figure 1C**).

**FIGURE 2:**
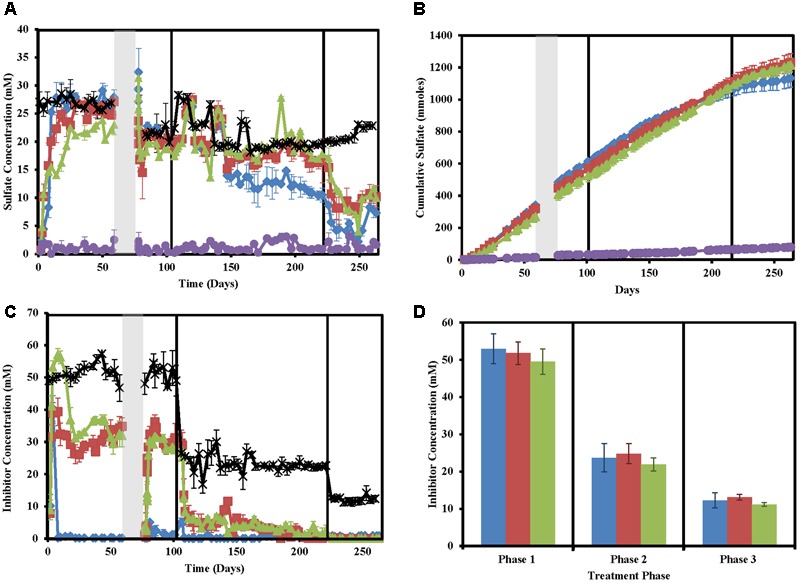
Sulfate and inhibitor concentrations in the column sets over time for nitrate-treated columns (blue diamonds/bars), chlorate (red squares/bars), perchlorate (green triangles/bars), no treatment (purple circles/bars), and influent (black stars). Phases of treatment are represented by boxes with the first being 50 mM, followed by 25 mM, and 12 mM. The shut in period is represented by a gray bar. All points are averages of triplicate columns with error bars representing SD of the triplicate samples except panel **(D)**. **(A)** Influent and effluent sulfate concentrations over time. **(B)** Cumulative sulfate concentrations over time. **(C)** Influent and effluent inhibitor concentrations over time. **(D)** Average inhibitor concentrations across each phase of treatment.

As expected, sulfate concentrations in treated columns decreased concomitantly with sulfide concentration increases (**Figures 2A,B**). However, sulfide and sulfate did not mass balance likely due to problems in accurately measuring sulfide and sulfide partitioning (Supplementary Figure S2). Despite large fluctuations in measured sulfide concentrations in the untreated columns, very little to no sulfate (0 ± 0 to 3.07 ± 0.52) was measured in these columns throughout the study indicating that total sulfate (22.32 ± 3.084 mM) was consistently consumed.

Influent inhibitor concentrations were very close to the goal concentrations of 50 mM (phase 1), 25 mM (phase 2), and 12.5 mM (phase 3, **Figure 2D**). Effluent inhibitor concentrations were significantly lower than influent concentrations indicating respiratory reduction of the inhibitors by the microbial community present in the columns (**Figure 2C**). Nitrate concentrations immediately fell to zero by the first sampling point after treatment (day 8) and stayed near zero throughout the entire study. Perchlorate required ∼8 days to build up to influent concentrations in the effluent and initially appeared to not be metabolized but by day 22 the effluent concentrations fell from an average of 50.0 ± 1.7 mM (all inhibitors) in the influent to 31.2 ± 0.7 mM perchlorate in the effluent. This coincides with the decrease in sulfide seen in respective columns (**Figure 2A**). Differential perchlorate concentrations between the effluent and influent indicate consumption of approximately 20 mM throughout phase 1 and phase 2 (residual perchlorate ∼5 mM). In phase 3 all perchlorate was consumed as would be expected based on the 20 mM consumed in phases 1 and 2, and the fact that only ∼12.5 mM perchlorate was used in the influent of this phase of the experiment. Unlike perchlorate, chlorate effluent concentrations in phase 1 averaged 39.3 ± 4.3 mM compared to an influent concentration of 53.0 mM. This indicates that chlorate is immediately reduced 10 mM, either biotically or abiotically. After this point, effluent chlorate concentrations continue to fall and similar reduction rates to perchlorate are seen throughout the other phases of treatment.

### Isotopic Analyses

**FIGURE 3:**
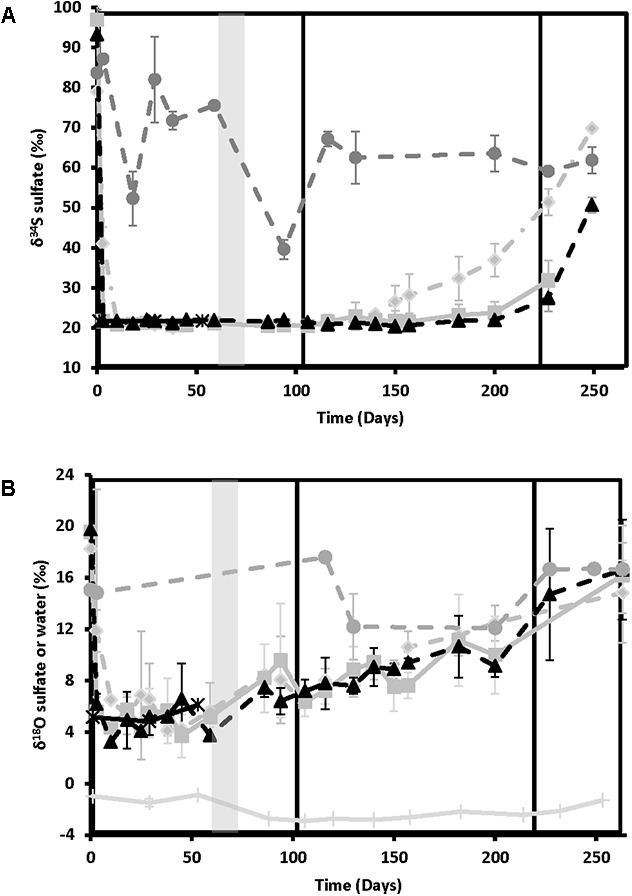
Isotopic ratios of the various treatments over time for nitrate-treated column effluent samples (light gray diamonds and dashed lines), chlorate (medium gray squares and solid lines), perchlorate (black triangles and dashed lines), no treatment (dark gray circles and dashed lines), and influent (black stars and solid lines). Phases of treatment are represented by boxes with the first being 50 mM, followed by 25 mM, and 12 mM. The shut in period is represented by a gray bar. **(A)** Variation in sulfur isotope ratios of dissolved sulfate with time. **(B)** Variation in oxygen isotopes of dissolved sulfate or water over time. All points are averages of triplicate columns with error bars representing SD of the triplicate samples.

**Figure 3** shows the sulfur isotope data for influent and effluent sulfate samples. The influent values remained constant throughout (δ^34^S = 21.2 ± 0.6‰, *n* = 23), providing a good baseline for comparison. Effluent values were high at day 0 for all treatments (86.8 ± 15.9‰, *n* = 9), indicating strong sulfate reduction, and remained elevated above influent values for the no treatment control. For the treated columns, effluent δ^34^S values decreased to match influent values as sulfide disappeared and sulfate concentrations rebounded (**Figure 1**). Nitrate-treated columns showed the most rapid rate of rebound in δ^34^S values, starting to increase above influent values on day 130, during the ∼25 mM treatment phase (21.8–22.6‰ in the different replicate columns), and reaching a high of 48.9–69.8‰ during the ∼12.5 mM treatment phase. In contrast, chlorate treatment results were slightly erratic in phase 2. One of the chlorate columns increased slightly above influent δ^34^S values as early as day 116 (23.5‰), with the second column increasing by day 182 (23.6‰) but chlorate δ^34^S did not increase notably in all columns until after chlorate influent concentrations were lowered to ∼12.5 mM (i.e., after day 221). Perchlorate columns were the last to increase above influent values, with one column increasing by day 182 (23.3 ‰) and the remaining columns increasing during the ∼12.5 mM treatment phase.

Oxygen isotopes of sulfate and water are shown in **Figure 3B**. Sulfate-oxygen isotope influent values were 5.1 ± 1.0‰ (*n* = 6), while water-oxygen values varied between -0.9 and -3.0‰, depending on the batch of San Francisco Bay water used. Effluent δ^18^O-sulfate was 18.6 ± 2.7‰ (*n* = 7) at day 0 and remained elevated above influent values in the no treatment control columns. In contrast to the sulfur isotopes, effluent sulfate-oxygen isotopes for the treated columns all showed a gradual increase in values above the influent values after the shut-in period in phase 1 (∼50 mM treatment concentrations) supporting ongoing sulfur cycling. These sulfate-oxygen isotopes continued to increase gradually throughout the experiment, reaching similar values to the no treatment control by day 263 (16.0 ± 2.7‰, *n* = 10, **Figure 3B**). It should be noted that both the absolute range of values and the reproducibility between individual columns for each treatment is lower for δ^18^O-sulfate than for δ^34^S; however, the observed trend remains clear.

### Microbial Community

**FIGURE 4:**
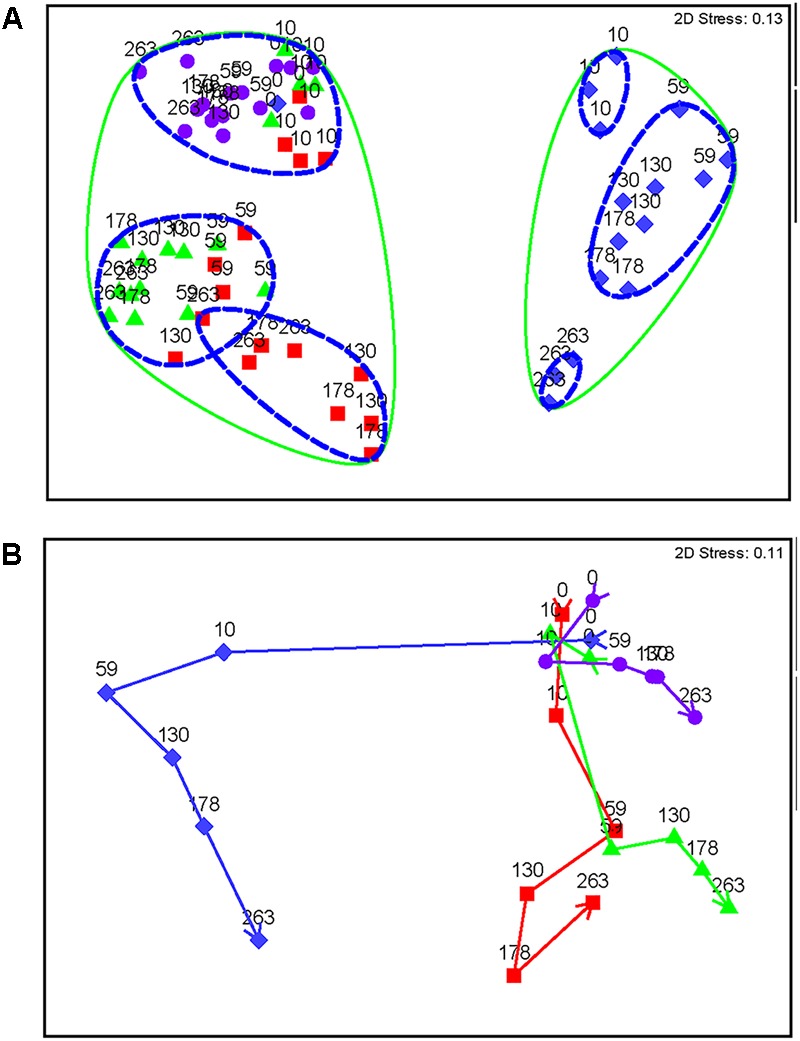
Non-metric multidimensional scaling plots of Bray Curtis similarity of standardized and square root transformed OTU data. Diamonds represent nitrate-treated columns, squares represent chlorate-treated columns, triangles represent perchlorate-treated columns, and circles represent untreated control columns. **(A)** All samples over time. Each point is labeled with sample time in days. Points that have 90% similarity or more to each other are circled with a solid line and points that are 92% similar or more are circled with a dashed line. **(B)** Means plot averaged by each day within each treatment. Arrows represent trajectory of the community over time. Stress values are an indication of how well the 2D representation fits the data with a stress of zero being a perfect representation and a stress value of 0.3 being a random representation of the data.

Nitrate-treated samples had a distinct community from all other treatments, grouping at 90% similarity (**Figure 4A**). Within this group, days 10 and 263 each grouped separately (92% similarity), while all other time-points grouped together (92% similarity), despite treatment concentration, which decreased over time (**Figure 4A**). Other treatments (chlorate, perchlorate, and no treatment) grouped together at 90% similarity. The untreated samples and pre-treatment (day 0) samples grouped with chlorate and perchlorate day 10 (92% similarity), which was before sweetening was observed in these treatments. All other perchlorate time-points grouped (92% similarity) with the chlorate day 59 and one replicate of chlorate day 130. All other chlorate-treated samples grouped together (92% similarity).

All treatments show a trajectory over time (**Figure 4B**). Untreated samples do change slightly temporally; however, the treated samples show a much stronger and more distinct change. Nitrate has a very strong change by day 10, while perchlorate- and chlorate-treated columns show a slight community change at this time-point (**Figure 4B** and Supplementary Figure S2). This corresponds with the geochemistry data that shows sweetening by day 10 in the nitrate-treated columns, while the (per)chlorate-treated columns lagged behind. By day 59, when all columns were sweetened, community structure in (per)chlorate-treated column samples reflected a stronger community change. Interestingly, the (per)chlorate columns showed similar trajectories to each other and the community change for nitrate was quite distinct.

**FIGURE 5:**
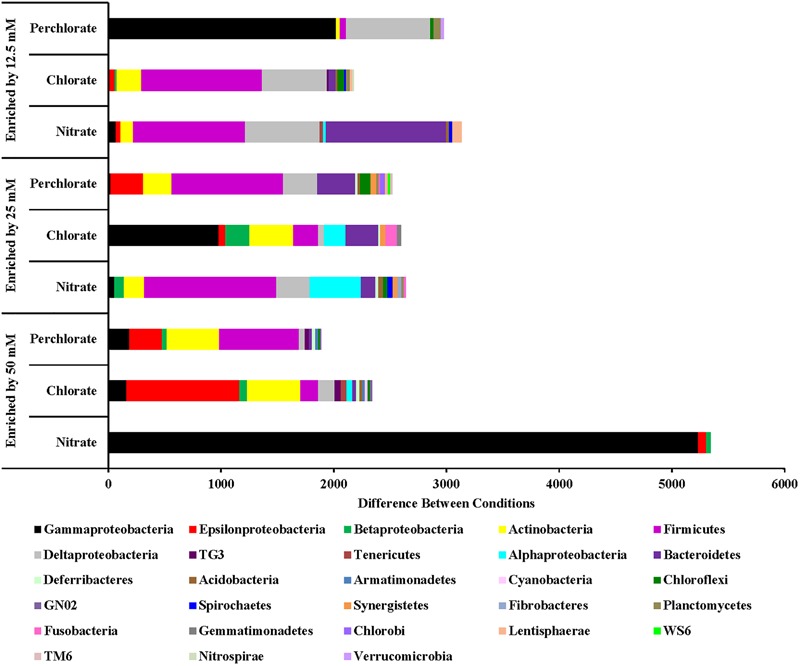
Phyla that are enriched by each treatment concentration compared to the previous concentration. Values are calculated by doing a similarity percentage (SIMPER) analysis of the top 10% of the difference between treatment concentrations for each treatment type on the standardized, square root transformed OTU data. The positive OTU differences are then summed by phyla (class for Proteobacteria) and represented as the “difference between conditions.”

For the nitrate treatment, the different treatment concentrations (phases) enriched for different organisms (**Figure 5**). Phase 1 of treatment enriched for *Proteobacteria* (*Gamma, Delta*, and *Beta*), *Bacteroidetes, Firmicutes*, and *Actinobacteria*. Many Proteobacteria (*Delta* and *Gamma*) families with members known to be involved with sulfur cycling are enriched including sulfate reducing *Desulfobacteraceae* (*Desulfosarcina* and unclassified), sulfate reducing *Nitrospiraceae* (*Nitrospira*), *Desulfobulbaceae* (*Desulfotalea, Desulforopalus, Desulfobubus*, and unclassified; sulfate reducing or sulfide oxidizing), and unclassified *Chromatiaceae* (sulfide and S° oxidation to sulfate). Genera and families containing known nitrate reducing organisms and fermenters were also enriched. *Nitrosomonas* (Class *Betaproteobacteria*) and unclassified *Flavobacteriaceae* (Phylum *Bacteroidetes*) contain members capable of denitrification, unclassified *Comamonadaceae* (Class *Betaproteobacteria*) contain members capable of nitrate reduction, and both *Carnobacteriaceae* (genus *Tricococcus*) and *Porphyromonadaceae* (unclassified genus) are known to contain fermenting organisms. Phase 2 of nitrate treatment also enriched for families of *Proteobacteria* containing sulfur cycling organisms (unclassified *Desulfobacteraceae, Desulfuromonadaceae* genus *Desulfuromonas*, unclassified *Chromatiaceae*, unclassified *Ectothiorhodospiraceae, Idiomarinaceae* genera *Pseudidiomarina* and *Idiomarina*, and *Thiotrichaceae* genus *Leucothrix*), including some of whom can potentially couple sulfide oxidation to nitrate reduction (*Campylobacteraceae* genus *Campylobacter*). Some of these (e.g., *Desulfobacteraceae* and *Desulfuromonadaceae*) may also contribute to the re-souring seen in the geochemical data during this treatment period. Families containing nitrate reducing organisms (*Alteromonadaceae* genera *Microbulbifer* and *Marinobacter, Alcanivoraceae* genus *Alcanivorax, Enterobacteraceae* genera *Pantoea* and *Erwinia, Idiomarinaceae* genera *Pseudidiomarina* and *Idiomarina*, and *Oceanospirillaceae* genus *Marinobacerium*) were also enriched. During treatment phase 3 some of the same nitrate reducing families were enriched but no further enrichment of known sulfur cycling families was observed compared to phase 2 of treatment.

**FIGURE 6:**
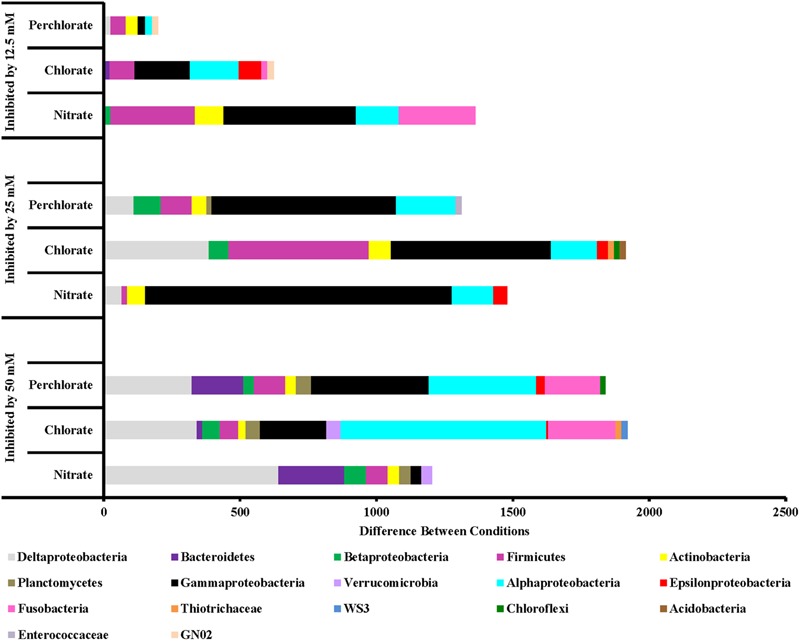
Phyla that are inhibited by each treatment concentration compared to the previous concentration. Values are calculated by doing a similarity percentage (SIMPER) analysis of the top 10% of the difference between treatment concentrations for each treatment type on the standardized, square root transformed OTU data. The negative OTU differences are then summed by phyla (class for Proteobacteria) and represented as the “difference between conditions.”

Chlorate and perchlorate treatments enrich for a wider diversity of phyla with *Proteobacteria* (*Epsilon* and *Gamma*), *Actinobacteria, Enterococaceae*, and *Firmicutes* being the most enriched (**Figure 6**). Families containing known PRB were enriched in the high-perchlorate treatment phases. *Helicobacteraceae* (*Sulfurimonas*) and *Campylobacteraceae* (*Arcobacter*), both *Epsilonproteobacteria* with known PRB members, were enriched during treatments phases 1 and 2 (Carlström et al., 2013; Barnum et al., 2018). *Gammaproteobacteria* enriched during the first phase of perchlorate treatment include *Shewanellaceae* (*Shewanella*), *Succinovibrionaceae* (*Succinivibrio*), *Vibrionaceae* (*Vibrio*), *Cardiobacteriaceae* (*Cardiobacterium*), and *Pseudomonadaceae* (*Pseudomonas*). None of these families includes a known PRB but *Pseudomonadaceae* and *Shewanellaceae* both contain representatives of known chlorate reducing organisms (Wolterink et al., 2002; Steinberg et al., 2005; Clark et al., 2013; Clark et al., 2016). The switch to the second phase of treatment enriches for a wider diversity of phyla, with *Firmicutes* being the most enriched in perchlorate-treated samples and *Gammaproteobacteria* being the most enriched in chlorate-treated samples including the families *Sedimentacolaceae* (*Sedimenticola*) and *Shewanellaceae* (*Shewanella*), which contain known chlorate reducing organisms (Clark et al., 2013; Carlstrom et al., 2015). During treatment phase 2, the *Deltaproteobacteria* (composed mostly, but not entirely, of SRB) were enriched in perchlorate-treated columns which is supportive of active sulfur redox cycling as suggested by the stable isotope data. In chlorate-treated columns families included *Desulfobacteraceae* (genera *Desulfobacter*, and unclassified) and *Desulfobulbaceae* (genera *Desulforhopalus, Desulfocapsa*, and unclassified). During treatment phase 3 in the perchlorate columns, the sulfate reducing families *Desulfovibrionaceae* (genus *Desulfovibrio*) and *Desulfobacteraceae* (Genera *Desulfobacter, Desulfobacterium*, and unclassified) were enriched as was the *Desulfobulbaceae* (Genera *Desulfocapsa* and unclassified).

Along with an enrichment effect on some organisms, all treatments also had observable inhibitory effects (**Figure 6**). Perchlorate treatment (phase 1) inhibited a variety of phyla including *Proteobacteria* (*Gamma, Delta, Epsilon*, and *Beta*), *Firmicutes, Actinobacteria, Chloroflexi, Enterococcaceae, Bacteroidetes, Defferibacteres, Fusobacteria*, and *Armatimonadetes*. Phase 2 of this treatment was also broadly inhibitory toward many of the same phyla while phase 3 of treatment appears to have inhibited *Proteobacteria* (*Alpha, Delta*, and *Gamma*), *Firmicutes*, and *Actinobacteria* to a lesser extent. Chlorate treatment showed a similar trend of broad inhibition at very high-treatment concentrations and less broad as the treatment concentration was lowered. Nitrate treatment also inhibited a range of different phyla including *Deltaproteobacteria* and *Bacteriodetes* (treatment phase 1), *Gammaproteobacteria* and *Alphaproteobacteria* (treatment phases 2 and 3) along with *Firmicutes* and *Fusobacteria* (treatment phase 3).

**FIGURE 7:**
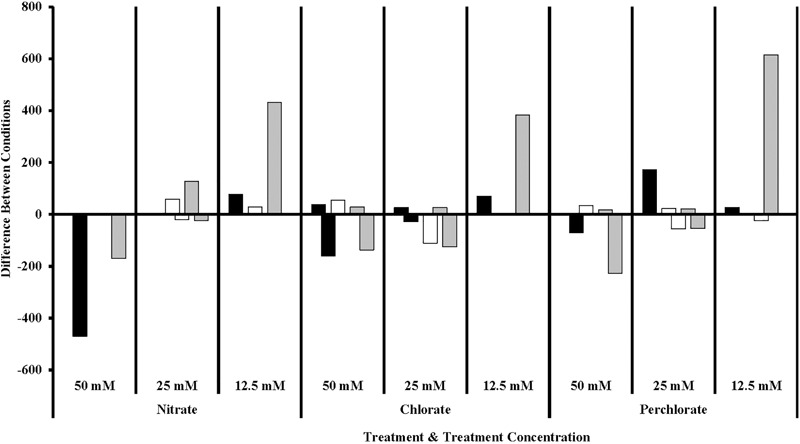
Sulfur cycling families of Delta Proteobacteria in different treatment concentrations compared to the previous concentration. Values are calculated by doing a similarity percentage (SIMPER) analysis of the top 10% of the difference between treatment concentrations for each treatment type on the standardized, square root transformed OTU data. The negative or positive OTU differences are then summed by sulfur cycling family and represented as the “difference between conditions.” The organisms identified as sulfate reducing organisms (gray bars) are members of *Desulfovibrionaceae, Nitrospiraceae, Syntrophecaceae*, and *Desulfobacteraceae*, the organisms identified as sulfide oxidizing organisms (black bars) are members of *Desulfobulbaceae*, and the organisms identified as sulfur reducing (white bars) organisms are members of *Desulfomonadaceae*.

Of particular interest is the inhibitory effect each treatment had on the sulfur cycling *Deltaproteobacteria* (**Figure 7**), which included known families that include SRB (*Desulfovibrionaceae, Nitrospiraceae, Syntrophacaceae*, and *Desulfobacteraceae*), *Desulfobulbaceae* (a family known for its member’s ability to oxidize sulfide to produce either sulfate or elemental sulfur although some members can alternatively perform sulfate reduction) and *Desulfomonadaceae* (family known to specifically reduce sulfur). All treatments in phase 1 inhibited known SRB relative to the non-amended control columns, preventing their return until phase 2 (nitrate-treated samples) or phase 3 (perchlorate- or chlorate-treated samples). High-nitrate treatment had the largest inhibitory effect on *Desulfobulbaceae*, a group within the *Deltaproteobacteria*, with (per)chlorate having a lesser effect. Unexpectedly, lower levels of perchlorate treatment (25 mM, phase 2) appeared to stimulate growth of this family. Sulfur reducing organisms are present in some of the treated samples but do not appear to play a significant role.

### Model Simulation

**FIGURE 8:**
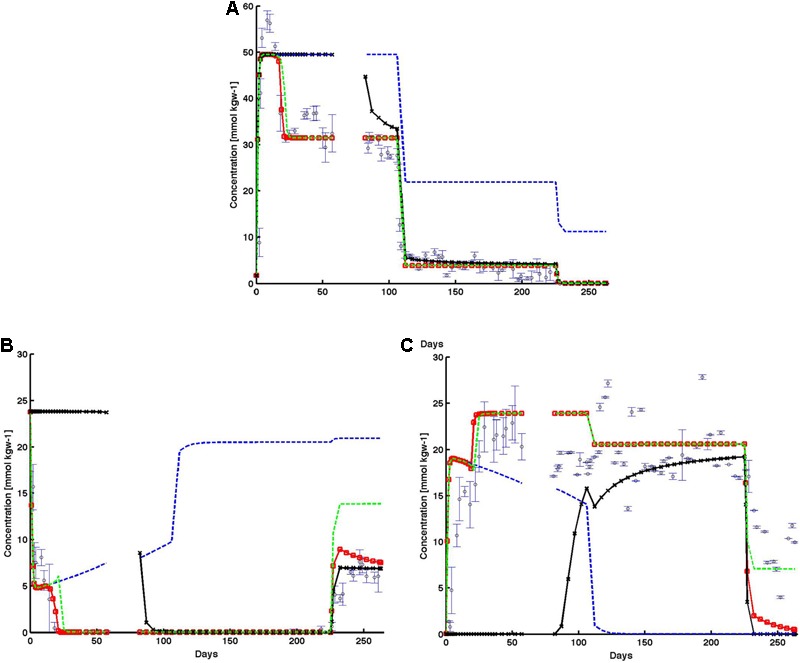
Observed (open black circles) and simulated **(A)** effluent perchlorate, **(B)** effluent sulfide, and **(C)** effluent sulfate from the base case (red lines), and the subsequent cases in which individual inhibition mechanisms were systematically removed: (1) no perchlorate inhibition (black lines); (2) no HPR (blue-dashed lines); and (3) no PRSO (green-dashed lines). The shut in period is represented by a breaks in the lines. All observed points are averages of triplicate columns and error bars represent SD of the triplicate values.

In the baseline case, the model reproduced the observed trends of effluent sulfate, sulfide, and perchlorate data (observed data – open blue circles, **Figure 8**). Data (**Figure 8A**) showed that perchlorate breakthrough and effluent concentration increased rapidly within 10 days to influent concentration values, before decreasing to around 30 mmol kgw^-1^ for the period before the shut in (day 57) representing a loss of ∼20 mmol kgw^-1^. When influent perchlorate concentration was decreased to 22 mmol kgw^-1^ starting day 106, effluent concentration rapidly decreased to 4 mmol kgw^-1^ at day 112 and remained at this concentration again representing a loss of ∼20 mmol kgw^-1^. During the final reduction of influent perchlorate concentration beyond day 225, no effluent perchlorate could be detected. Observed effluent sulfide (**Figure 8B**) concentration rapidly decreased to zero within the first 20 days after perchlorate injection began. Thereafter, throughout the experiment, when effluent perchlorate was detected, no sulfide was observed in the effluent (**Figure 8B**). The exception was during the final phase when influent perchlorate concentration was reduced to 11 mmol kgw^-1^. As a result, no effluent perchlorate was detected, and effluent sulfide concentration increased to ∼7 mmol kgw^-1^ during the final days of the experiment, suggesting a total electron acceptor consumption of ∼18 mmol kgw^-1^. As the electron accepting capacity of perchlorate (8 e^-^) for complete reduction to chloride is identical to that of sulfate to sulfide, an approximation would suggest that perchlorate is acting as a preferential electron acceptor for the competitive exclusion of SRB. In support of this, the observed effluent sulfate trend (**Figure 8C**) was inversely related to effluent sulfide concentration. During the initial effluent sulfide reduction, the effluent sulfate concentration increased correspondingly and returned to influent values thereafter (**Figure 8C**). During the final phase, when effluent sulfide increased, effluent sulfate correspondingly decreased to ∼7 mmol kgw^-1^. The model (red line) was able to reproduce the rapid decrease (increase) in effluent sulfide (sulfate) during the first 20 days. The model also showed zero sulfide production throughout the remaining time period until the final phase. During the final phase, modeled sulfide also captured the rising trend in observed effluent sulfide. Simulated effluent sulfate captured the decreasing observed sulfate trend but was lower than the observed trend by ∼2–8 mmol kgw^-1^.

Comparison of the alternative cases against the baseline simulation (red line) showed the relative impact of each perchlorate inhibition mechanism: direct chemical inhibition (black line), HPR (dashed blue line), and perchlorate reduction linked to sulfide oxidation (PRSO, dashed green line) on effluent chemistry behaviors (**Figure 8**). In the absence of direct inhibition of SRB by perchlorate (case 2, black line in **Figure 8**), sulfate reduction continued and simulated effluent sulfate remained at zero prior to the 14-day shut-in period at day 57. During the same period of time, simulated effluent sulfide remained high at ∼24 mmol kgw^-1^ (complete conversion of influent sulfate to sulfide). After the shut-in period, simulated sulfide decreased to zero and remained at zero till day 225. Correspondingly, immediately after the shut-in, simulated effluent perchlorate decreased from ∼45 to around 30 mmol kgw^-1^ (∼day 110). Thereafter, simulated perchlorate trend in this case followed that of the baseline simulation (red line, **Figure 8A**). Simulated effluent sulfate (black line) increased rapidly after the shut-in, from 0 to ∼20 mmol kgw^-1^ (right before the final reduction of influent perchlorate). Beyond day 225, in response to the final reduction in influent perchlorate concentration, simulated effluent sulfide increased to ∼7 mmol kgw^-1^, while effluent sulfate decreased to zero.

For case 3 (dashed blue lines, **Figure 8**), where the role of competitive inhibition of SRB by heterotrophic PRB is removed, the rate of perchlorate reduction becomes negligible, as shown by effluent perchlorate concentration matching influent values throughout the simulation timeframe (**Figure 8A**). The inhibitory impact of perchlorate was able to reduce sulfate reduction rates particularly in the first ∼120 days when perchlorate concentration was ∼50 mmol kgw^-1^. Subsequently, as influent perchlorate concentration was lowered, sulfate reduction rate increased, and sulfate (**Figure 8C**) was completely converted to sulfide (**Figure 8B**).

For case 4, where the role of sulfide oxidation coupled to perchlorate reduction by PRSO is removed (dashed green lines, **Figure 8**), simulation results remained similar to the baseline case (dashed red line with red circles, **Figure 8**) throughout much of the simulation. The exceptions are: (1) initial drop in effluent sulfide (first ∼20 days), and (2) the last phase in which influent perchlorate concentration was reduced to ∼12 mmol kgw^-1^. In case 4, simulated effluent sulfide took longer than the baseline case to drop to zero. In fact, simulated effluent sulfide in case 4 experienced a little rebound before dropping to zero. This showed that in combination with the perchlorate inhibition mechanism, the PRSO mechanism further reduced sulfide to levels low enough for heterotrophic PRB to set in. The PRSO mechanism plays a dominant role in last phase, as revealed by the effluent sulfide rebound (**Figure 8**). In the absence of PRSO, effluent sulfide concentration increased to as high as ∼15 mmol kgw^-1^ (compared to 7 mmol kgw^-1^ in the base case).

## Discussion

Previous packed bed column studies have clearly demonstrated the effectiveness of nitrate as both a souring inhibitor (Vance et al., 2005; Gieg et al., 2011) and a souring reversal agent (Hubert and Voordouw, 2007) but (per)chlorate was only studied in a column system as a souring inhibitor (Engelbrektson et al., 2014). In this study, we tested (per)chlorate as a sweetening agent in a previously soured system.

### Geochemistry

High levels (50 and 25 mM) of all treatments are completely effective, although perchlorate takes longer to completely reverse the souring than the other two treatments (nitrate or chlorate). The slower effect by perchlorate is likely due to the relative rarity and lower abundances of perchlorate reducing organisms in the environment versus nitrate reducers, which are ubiquitous. Chlorate can react abiotically with both the iron and the sulfide in the system thus explaining ∼40% of the treatment disappearing immediately inside the column despite the rarity of chlorate reducers in a bay water system (Engelbrektson et al., 2014). However, the nitrate-treated columns are quick to re-sour when treatment levels are decreased to 25 mM and the other two treatments did not. Presumably this is due to the fact that (per)chlorate persists in the effluent at low but measurable concentrations during treatment phase 2 while measurable nitrate is not seen in the effluent. However, this does not explain why 50 mM nitrate is effective while column effluent concentrations are also zero. It is possible that nitrite was present in the columns when they were treated with the high level of nitrate, due to incomplete nitrate reduction. However, because nitrite, which was not measured in this experiment, chemically reacts with reduced iron (Van Cleemput and Baert, 1983; Dhakal et al., 2013), we would not expect to measure nitrite in columns containing iron rich bay sediment. It is also possible that there is stratification in the columns, with most or all sulfate reduction occurring close to the injection point of the column, thus the electron donor and acceptors would also be consumed at the inflow of the column, resulting in very little activity at the outflow of the column. If this is the case, then low-effluent concentrations of inhibitor may not reveal the true effects early in the column. Despite the mechanism, nitrate-treated columns do indeed re-sour at lower concentrations than (per)chlorate-treated columns.

### Isotopes

Overall, the δ^34^S values of the effluent sulfate matched the trends seen in effluent sulfide and sulfate with the highest δ^34^S values corresponding to the greatest degree of microbial sulfate reduction. This process is well known to fractionate sulfur isotopes as SRB preferentially use the lighter isotope (^32^S), leaving the residual sulfate relatively enriched in ^34^S (Kaplan and Rittenberg, 1964; Brüchert, 2004; Brunner and Bernasconi, 2005). The sulfur isotopes do not add additional insight into the sulfur cycling evidenced by sulfate and sulfide concentrations, although they do suggest a very minor degree of net sulfate reduction from day 116 onward in one out of three of the chlorate columns not apparent from the mean effluent chemistry profiles in **Figures 1, 2**.

In contrast, the sulfate-oxygen isotope data do add insight into the complexity of the ongoing sulfur cycling in the columns (Hubert et al., 2009). During microbial sulfate reduction, numerous studies have shown that sulfate-oxygen can reach apparent equilibration with water-oxygen, becoming enriched in ^18^O by up to 23–29‰ compared with water-oxygen (Fritz et al., 1989; Brunner et al., 2005; Zeebe, 2010). The primary mechanism which affects the sulfate-oxygen signature is commonly considered to be rapid equilibration of the oxygen in sulfite with water-oxygen, which will leave the sulfite enriched in ^18^O compared with the water (Brunner et al., 2012; Müller et al., 2013; Wankel et al., 2014). Sulfite forms as an intermediate during microbial sulfate reduction and during the oxidation of reduced sulfur compounds to sulfate. Microbial sulfate reduction consists of multiple enzymatic steps, many of which are reversible. In the situation where sulfite reduction to sulfide occurs at a slower rate than sulfate reduction to sulfite, then equilibration of sulfite with water and back flux of the sulfite to sulfate will alter the δ^18^O-sulfate of the cell-external residual sulfate pool. As applied to the column experiments, this could help to explain the small shifts in δ^18^O-sulfate seen when δ^34^S changes are minimal or unresolvable within the reproducibility of the technique (i.e., extremely low-net fluxes of sulfate reduction). This situation could conceivably occur when (per)chlorate concentrations (or nitrite) are high enough to largely inhibit sulfate reduction, leaving a reduced population of SRB very weakly metabolizing (perhaps even within micro-niches or the protection of biofilms within the column) until conditions are more favorable for them once again. As the column experiments progress further, conditions become more favorable for the SRB and sulfate reduction fluxes increase, resulting in larger shifts in δ^18^O-sulfate and also in δ^34^S.

An alternative explanation for the sulfate-oxygen data involves a balance of microbial sulfate reduction and re-oxidation of sulfide all the way to sulfate. Complete re-oxidation to sulfate would cause no shift in the δ^34^S signature but would alter δ^18^O-sulfate by equilibration of a sulfite intermediate with water and/or incorporation of water-oxygen (and/or oxygen from AMP) into the sulfate (Turchyn and Schrag, 2006; Hubbard et al., 2009; Hubbard et al., 2014). All treatments enriched for phyla known to include organisms capable of sulfide oxidation. This metabolism has been demonstrated in both pure cultures and communities for nitrate (Hubert et al., 2009). In contrast there is no known PRB in pure culture capable of this metabolism, although it is thermodynamically favorable (Δ*G^0^’* = -783 kJ mol^-1^ ClO_4_^-^) and studies with ill-defined perchlorate reducing communities have previously offered some empirical support (Ju et al., 2007, 2008; Gregoire et al., 2014; Mehta-Kolte et al., 2017).

At the moment, no reactive transport models capture sulfate-oxygen dynamics; therefore these mechanisms cannot be constrained by our data and modeling approach. The sulfate-oxygen data does, however, show evidence for a very minor degree of microbial sulfate reduction occurring throughout most of the experiment, and hints at possible cryptic sulfur cycling not shown by concentration data or sulfur isotopes. The sustained (if low) SRB activity helps to explain the persistence of SRB in the treatment columns, allowing there to be a population present to take over when inhibitor concentrations are lowered enough that excess donor is available for sulfate reduction. Alternatively these SRB could persist by relying on a different lifestyle during these periods, such as fermentation.

### Microbial Community

The microbial community data observed supports the measured geochemistry throughout the column study. SRB are inhibited at high levels of treatment and return when columns begin to re-sour, which is expected. Families known to contain PRB are enriched under those treatments. Untreated column samples are most similar to the initial soured samples and each treatment has a unique trajectory over time and treatment concentration, with (per)chlorate treatments sharing a similar trajectory. It is not surprising that perchlorate and chlorate treatments would enrich for similar organisms as all perchlorate reducing organisms can also reduce chlorate (Logan et al., 2001; Coates and Achenbach, 2004; Bardiya and Bae, 2011). Richness (Supplementary Figure S3) appears to be relatively unaffected by treatments indicating the lack of an overall inhibitory effect on the community and points to the specificity of the individual treatment.

### Reactive Transport Modeling

A reactive transport model of perchlorate treatment was also developed to complement the experiment. The base case simulation captured the temporal patterns of the effluent chemical species. Subsequent simulations, in which individual inhibition mechanisms were systematically removed, elucidated the relative role that each inhibition mechanism played at the different phases of the experiment.

Modeling results highlight the importance of perchlorate toxicity and bio-competition between PRB and SRB as key de-souring mechanisms before the shut-in (with perchlorate toxicity playing a more dominant role). The finding is similar to the study conducted by Wu et al. (2017). In fact, the current study can be viewed as an extended version of the Wu et al. (2017) study (with shut in and subsequent reduction in influent perchlorate concentration). In this study, the subsequent reduction in perchlorate concentrations brought about an interesting switch in the dominating mechanism: from perchlorate toxicity to bio-competition between PRB and SRB. In all, modeling in this study indicates that the absence of HPR brought about the greatest deviation from the base case simulation, suggesting bio-competition between PRB and SRB as a dominant mechanism of the sulfate reduction control. This finding is not surprising since HPR is the pathway through which PRB derive enough energy for growth. When this mechanism was removed, the PRB population was unable to establish themselves to compete effectively against the already established SRB population. As a result, mortality continued to reduce the population, along with the PRSO rates, while at the same time, SRB continued to exert dominance. The results are also consistent with an earlier reactive transport modeling study (Cheng et al., 2016). Sulfide oxidation was shown through modeling to be important during the initial and the final phases of treatment. Interestingly, simulation results fully complement the experimental findings.

Together, the geochemistry, microbial community, and modeling results reveal the relative role of each mechanism during the different phases of the experiment. In phase 1, direct inhibition of sulfate reduction by perchlorate and PRSO reduced the sulfide concentrations significantly enough for the hPRB mechanism to begin. Next, the hPRB mechanism drastically reduced the effluent perchlorate concentration, causing competition between perchlorate reducers and sulfate reducers for dissolved organic carbon to continue to limit sulfate reduction (and sulfide production). However, as influent perchlorate concentrations decreased, sulfate reduction rebounded. PRSO played a dominant role in reducing effluent sulfide concentration during the final phase, when influent perchlorate concentration is the lowest. The results highlight the interactive effects, which are otherwise hard to tease out, of the respective inhibition mechanisms at the various phases of the experiment, and the versatility of perchlorate as an inhibitor at different dosages.

## Author Contributions

AE and JC conceived and planned the experiments. AE was the primary researcher who performed the column study with the sampling assistance of YJ. AE did the majority of the geochemical and microbial community analyses. YC, BA, and JA-F created the reactive transport model. LT, PH, and GA isolated DNA, ran the phylochip, and performed some of the microbial community analyses. CH, A-LG, and MC performed the isotopic analyses. AE and JC wrote the manuscript with assistance from CH, YC, and BA.

## Conflict of Interest Statement

The corresponding author has an IP submission based on souring control. The remaining authors declare that the research was conducted in the absence of any commercial or financial relationships that could be construed as a potential conflict of interest.

## Footnotes

1 https://www.osha.gov/SLTC/hydrogensulfide/hazards.html
