# Supplementary material for: Attenuating Sulfidogenesis in a Soured Continuous Flow Column System With Perchlorate Treatment
Source: Front Microbiol. 2018 Jul 26;9:1575. doi: 10.3389/fmicb.2018.01575 (PMC6094985; doi:10.3389/fmicb.2018.01575)
Supplement: Supplementary file 1 [file Table_1.DOCX]

**Table S1 | Details about the treatment phases.** Numbers of days in each phase and the goal treatment concentration for each treatment phase.

| Treatment Phase | Days | Number of Days | Treatment Concentration |
| --- | --- | --- | --- |
| Phase 1 (Part 1) | 0-58 | 58 | 50 mM |
| Shut In | 59-78 | 21 | None (No Flow) |
| Phase 1 (Part 2) | 79-102 | 23 | 50 mM |
| Phase 2 | 103-221 | 118 | 25 mM |
| Phase 3 | 222-263 | 41 | 12.5 mM |
