# Supplementary material for: Attenuating Sulfidogenesis in a Soured Continuous Flow Column System With Perchlorate Treatment
Source: Front Microbiol. 2018 Jul 26;9:1575. doi: 10.3389/fmicb.2018.01575 (PMC6094985; doi:10.3389/fmicb.2018.01575)
Supplement: Supplementary file 3 [file Table_3.DOCX]

**TABLE S3 | Aqueous species concentrations in Initial Water (IW) and Amendment Water (AW).** Chemical concentrations in IW and AW used as initial and amendment conditions respectively in the CRUNCHTOPE simulations for the perchlorate treatment case described.

| Species | Initial Water (IW)  (mmol/kgw) | Amendment Water (AW)  (mmol/kgw) |
| --- | --- | --- |
| pH | 7.0 | 7.0 |
| Na(I) | 475.7 | 475.7 |
| Mg(II) | 53.6 | 53.6 |
| Ca(II) | 10.4 | 10.4 |
| Fe(II) | 0.0 | 0.0 |
| NH_4_(I)* | 15.0 | 15.0 |
| Cl(-I) | 428.6 | 428.6 |
| SO_4_(-II) | 23.89 | 23.89 (Days 1 -57) |
|  |  | 0.0 (Days 57 - 77) |
|  |  | 23.89 (Days 77 - 106) |
|  |  | 20.61 (Days 106 -225) |
|  |  | 21.02 (Days 225 -263) |
| HCO_3_(I) | 8.8 | 8.8 |
| DOC | 40.0 | 40.0 |
| S(-II) | 0.0 | 0.0 |
| ClO_4_(-I) | 0.0 | 49.5 (Days 1 -57) |
|  | - | 0.0 (Days 57 - 77) |
|  | - | 49.5 (Days 77 - 106) |
|  | - | 21.9 (Days 106 -225) |
|  |  | 11.2 (Days 225 -263) |
