# Supplementary figures and images for: Attenuating Sulfidogenesis in a Soured Continuous Flow Column System With Perchlorate Treatment
Source: Front Microbiol. 2018 Jul 26;9:1575. doi: 10.3389/fmicb.2018.01575 (PMC6094985; doi:10.3389/fmicb.2018.01575)

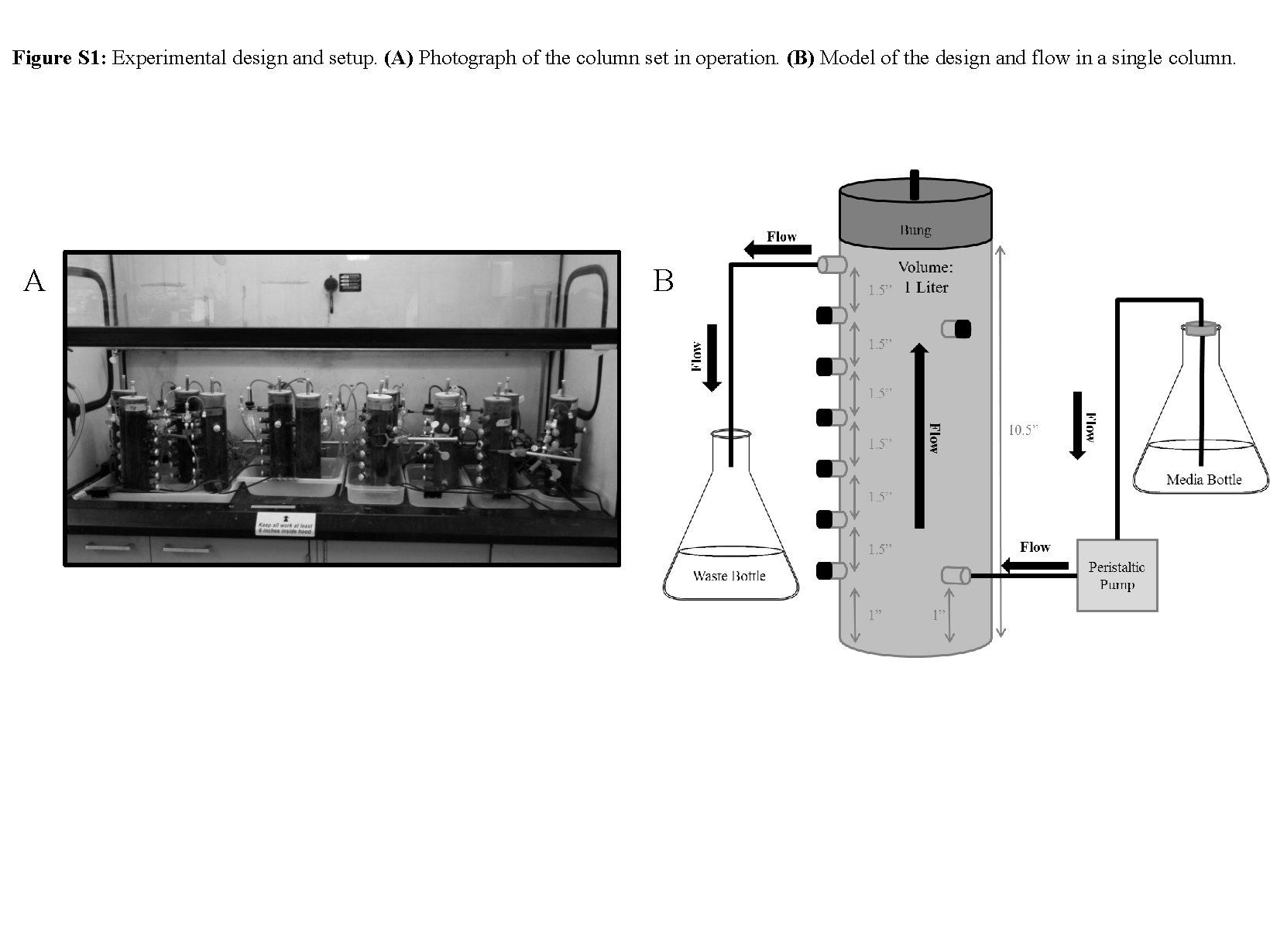

Supplement: Supplementary file 4 [file Image_1.TIFF]

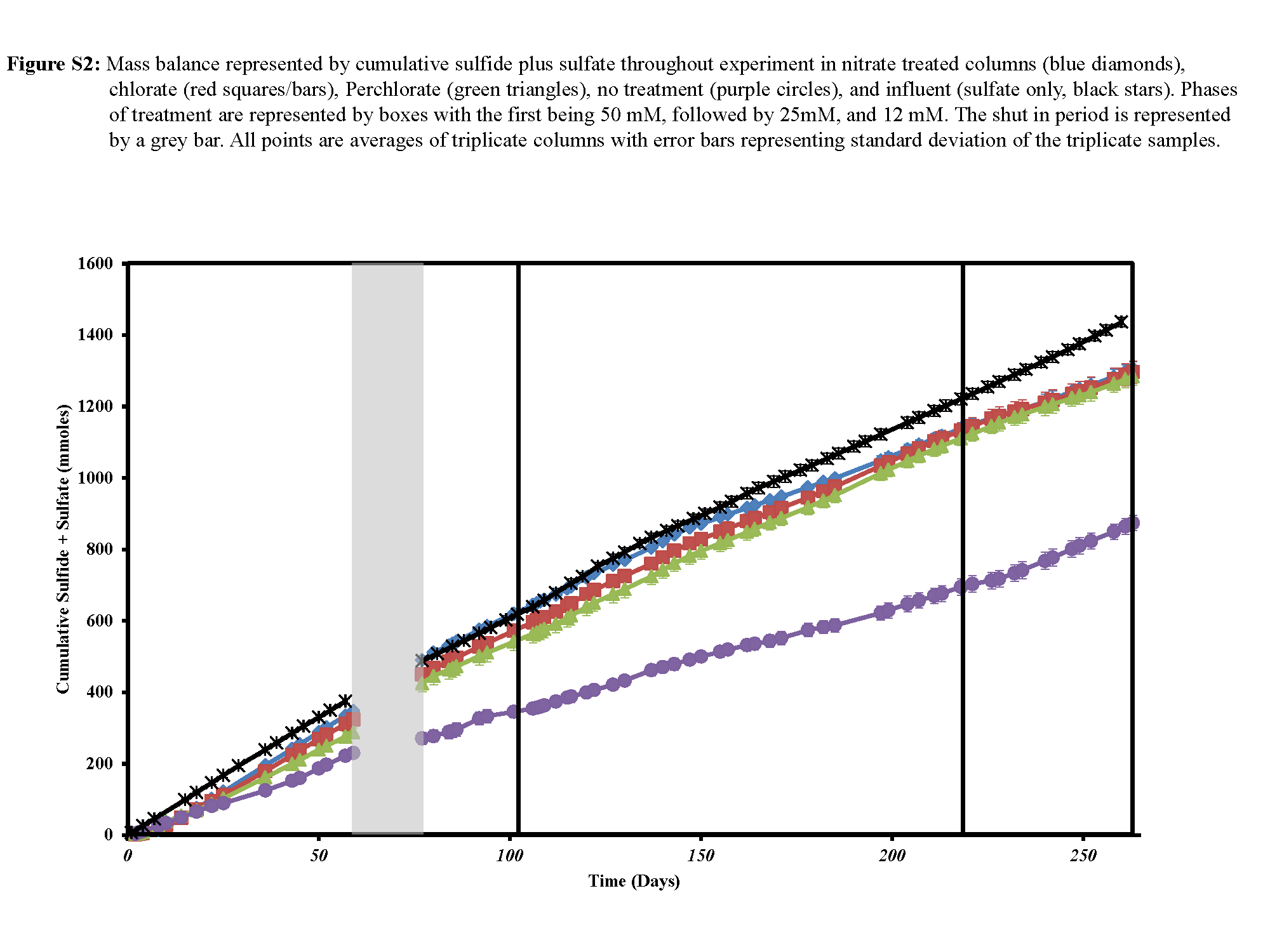

Supplement: Supplementary file 5 [file Image_2.TIFF]

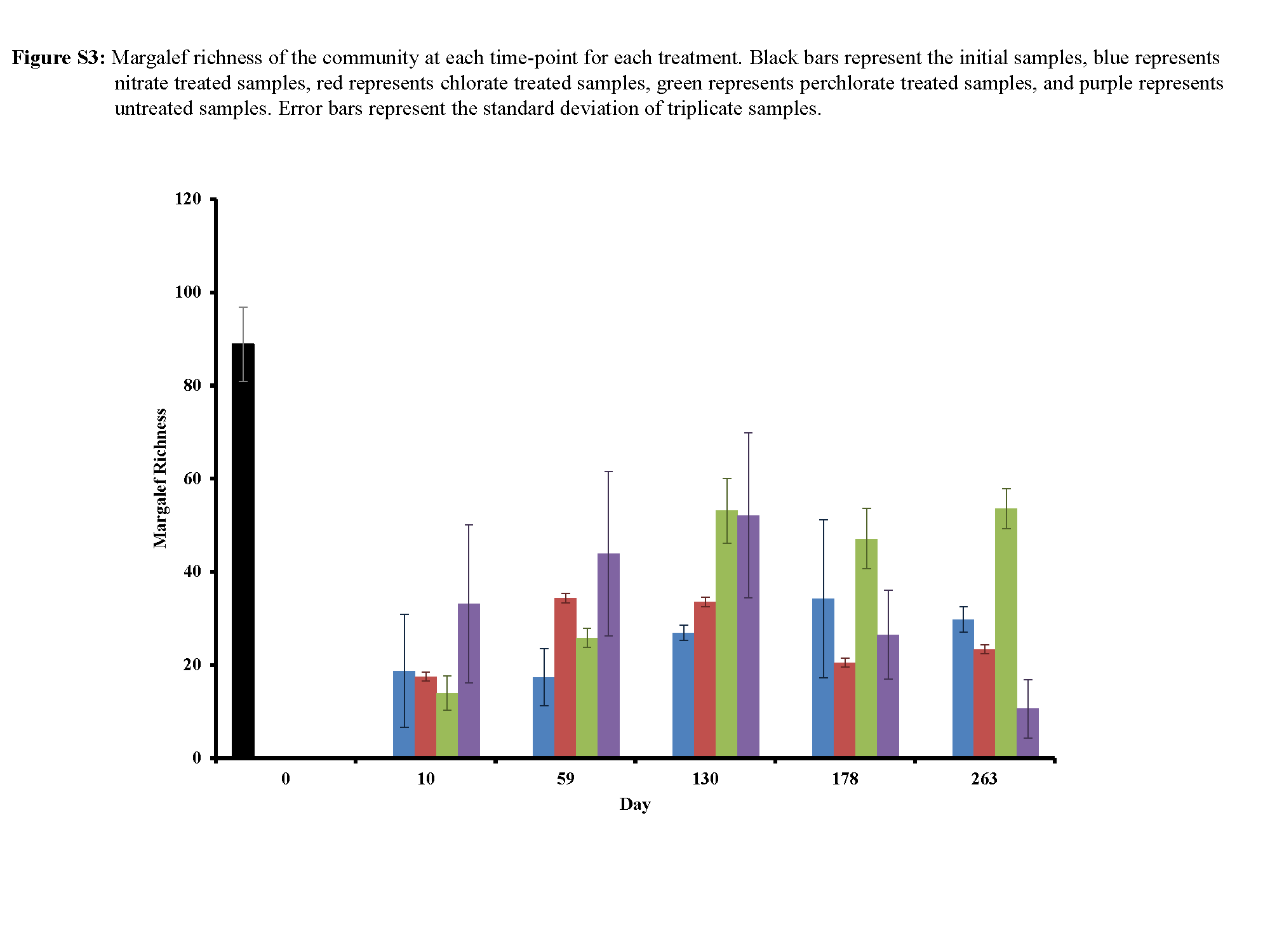

Supplement: Supplementary file 6 [file Image_3.TIFF]
